# Prevalence and relevant factors of positive RF in brucellosis patients with arthralgia

**DOI:** 10.1371/journal.pntd.0009749

**Published:** 2021-09-20

**Authors:** Siwen Zhang, Jing Hu, Shuqi An, Mujinyan Li, Fande Li, Peng Zhang, Xiangyi Zhang, Huixin Yang, Taijun Wang, Jingjing Luo, Fangfang Hu, Jiashuo Liu, Qing Zhen

**Affiliations:** 1 Department of Epidemiology and Biostatistics, Key Laboratory of Zoonosis, Ministry of Education, School of Public Health, Jilin University, Changchun, China; 2 The Sixth People’s Hospital of Shenyang, Shenyang, China; Universidad Peruana Cayetano Heredia, PERU

## Abstract

**Background:**

Brucellosis is a critical zoonotic disease in the world, it is the non-specific arthralgia that make brucellosis patients easily misdiagnosed as rheumatoid arthritis (RA) in endemic regions. Elevated rheumatoid factor (RF) is an essential indicator of RA, and the RF in brucellosis patients is significantly higher than healthy people. Therefore, this study further explored the distribution of RF and the relevant factors of the RF positivity in brucellosis patients with arthralgia, in order to strengthen the recognition of physicians for brucellosis patients with RF positivity, especially in brucellosis-endemic areas, so as to avoid misdiagnosis and untimely treatment that may lead to malignant outcomes.

**Methodology and principal findings:**

The medical records of all 572 brucellosis inpatients were collected in the Sixth People’s Hospital of Shenyang, China from 2015 to 2016. After excluding 106 patients without arthralgia, 5 patients who unwilling to perform RF testing and 16 patients with diseases that may affect RF, 445 brucellosis inpatients with arthralgia were involved in this retrospective cross-sectional study. 143 (32.1%) patients with RF >10 IU/ml were classified into the RF positive group, with an average level of 16.5[12.2, 34.7] IU/ml, of which 45 (10.1%) patients were high-positive with RF >30 IU/ml. Multivariate logistic regression model was used to further analyze the relevant factors of the RF positivity and found that age, wrist joint pain and elevated C-reactive protein (CRP) were positively associated with RF positivity, with OR of 1.02 (*P* = 0.024), 8.94 (*P* = 0.008) and 1.79 (*P* = 0.019), respectively.

**Conclusion:**

The prevalence of positive RF in brucellosis patients with arthralgia was critical, nearly one-third of patients had RF positive. Elderly men brucellosis patients with arthralgia, wrist joint pain and elevated CRP were at high risk of positive RF. It is reminded that physicians should focus on differential diagnosis during clinical diagnosis and treatment, especially in brucellosis-endemic regions.

## Author summary

Brucellosis is a highly contagious zoonosis caused by *Brucella* spp., which compromises to organs and systems, causing non-specific symptoms such as fever, headache, sweating, fatigue, myalgia and arthralgia. Similarly, patients with rheumatoid arthritis (RA) may also have the above non-specific symptoms. It is precisely because of the non-specificity and similarity of symptoms that brucellosis patients were easily misdiagnosed and failed to receive timely treatment, resulting in neurosis, chronic fatigue syndrome, endocarditis and other adverse outcomes. However, rheumatoid factor (RF) is an essential indicator of RA, and the RF in brucellosis patients is significantly higher than healthy people. In order to strengthen the recognition of physicians for brucellosis patients with RF positivity, we conducted this research and found that the prevalence of positive RF in brucellosis patients with arthralgia was common and critical. Elderly men brucellosis patients with arthralgia, wrist joint pain and elevated CRP were at high risk of positive RF. It is reminded that physicians should pay attention to the possibility of brucellosis during clinical diagnosis and treatment, especially in brucellosis-endemic regions, which had certain clinical significance.

## Introduction

Brucellosis caused by *Brucella* species is one of the most common zoonotic diseases in the world [1,2]. It remains a critical public health problem, with more than 500,000 new cases annually all over the world [3]. It is significant to pay attention to human brucellosis, in light of its great harm to the health of the population and the social economy, especially in some high-risk regions, such as the Mediterranean basin, the Middle East, and Central and South America [4,5]. Patients with brucellosis have fever, headache, sweating, fatigue, myalgia, arthralgia, hepatosplenomegaly and other manifestations [4,6], among which arthralgia is the most common clinical manifestation, which occurs in majority of patients and involves various parts of the skeletal system [7–10]. In addition, researches in clinical practice have found that fever and arthralgia in brucellosis patients were similar to the clinical manifestations of rheumatoid arthritis (RA), juvenile idiopathic arthritis, sarcoidosis and other diseases with arthralgia [11–13]. Therefore, it is the non-specific clinical manifestations that lead to the misdiagnosis and untimely treatment of brucellosis at the initial diagnosis [8,14]. It has previously been observed that 62.5% brucellosis patients are misdiagnosed at the first diagnosis [15]. Another research also found more than half of brucellosis patients were misdiagnosed as other diseases [8]. However, timely diagnosis and treatment have a pivotal role in preventing chronic of brucellosis. Chronic brucellosis not only causes malignant complications such as neurosis, chronic fatigue syndrome, endocarditis and adverse pregnancy outcomes, but also results in damage to the skeletal muscle system, difficulty in walking and even paralysis [14,16–18], which will reduce the patient’s quality of life and bring a significant financial burden.

Rheumatoid factor (RF) is an autoantibody against the fragment crystallizable portion of IgG [19]. Elevated RF is essential for the diagnosis and prediction of RA, especially high-positive RF that refers to three times the upper limit of normal, and RF can be found in 70%-80% of patients with RA [20–22]. However, elevated RF was also detected in the healthy elderly, as well as in some autoimmune diseases, such as systemic lupus erythematosus and Sjogren’s syndrome [23–26]. Besides, increasing researches shown that RF was positive in some infectious diseases, such as viral hepatitis, acquired immunodeficiency syndrome (AIDS) and tuberculosis [21,27–29]. A case-control study evaluating the rheumatologic laboratory markers of 49 brucellosis patients found that 15 (30.6%) patients were RF positive, which was significantly higher than healthy control people [30]. However, the research was limited to the small sample size, and it tended to focus on the positive rate of RF in brucellosis patients rather than the distribution level of RF and relevant factors of the RF positivity. Up to now, far too little attention has been paid to relevant factors of the RF positivity in brucellosis patients.

Therefore, we further explored the distribution of RF and the relevant factors of the RF positivity in brucellosis patients with arthralgia, in order to strengthen the recognition of physicians for brucellosis patients with arthralgia and RF positivity, especially in brucellosis-endemic areas, so as to avoid misdiagnosis and untimely treatment that may lead to malignant outcomes.

## Methods

### Ethics statement

The study protocol was approved by the bioethical committee at the Sixth People’s Hospital of Shenyang (20141009-SY12) and abided by the declaration of Helsinki principles. All patients or the respective parent of a minor signed an informed consent.

### Study population

We conducted a retrospective cross-sectional analysis of all brucellosis inpatients in the Sixth People’s Hospital of Shenyang, China from 2015 to 2016. In the present analysis, the medical records of all 572 brucellosis inpatients were collected, of which 466 brucellosis inpatients with arthralgia were selected as study population. We further excluded 5 patients who unwilling to perform RF inspection and 16 patients with diseases that may have a certain effect on RF, including viral hepatitis (9 cases), fatty liver (5 cases), rheumatic fever (1 case) and RA (1 case) [22,27–29,31,32]. And 445 brucellosis patients with arthralgia were eventually involved in this study.

### Measurements and variables

Brucellosis was diagnosed according to the epidemiological history, clinical manifestations, the isolation of *Brucella* spp. and serological examination. And clinical manifestations included fever, hyperhidrosis, fatigue, muscle and arthralgia, etc. Positive serological examination meant that the serum agglutination test (SAT) titer ≥1:100 (or the disease course lasted for more than one year and the SAT titer ≥1:50) [33]. And all 572 patients with brucellosis in this study included both clinically confirmed cases (epidemiological history and clinical manifestations were positive, symptoms were relieved after treatment, but the SAT results and *Brucella* spp. isolation were negative) and laboratory confirmed cases (epidemiological history, clinical manifestations and laboratory tests were all positive). Arthralgia was diagnosed based on the patient’s response to “Have you ever had any symptoms of joint pain?” on admission, mainly including spinal pain, knee pain, hip joint pain, shoulder pain, wrist joint pain, sacroiliac joint pain, ankle joint pain and toe joint pain.

Patients were divided into two groups for analysis according to the titer of RF detected by Latex Immunoturbidimetric Assay (BIOSINO, Beijing, China), where patients with RF >10 IU/ml were classified into the RF positive group, and patients with RF ≤10 IU/ml were classified into the RF negative group [34].

We selected general characteristics related to brucellosis for analysis, including demographics (gender, age and occupation), personal characteristics (i.e., past history of brucellosis, medication history, contact history, exposure method), clinical manifestations (i.e., clinical phase, fever, sweating, arthralgia), laboratory indicators (i.e., SAT, blood culture, aspartate aminotransferase (AST), C-reactive protein (CRP)). According to whether the patient was exposed to risk factors and the types of risk factors, we classified occupation into four groups: farmer and herdsman, veterinarian, processing staff (workers who slaughtering, processing or selling meat products, may contact with animal and their products) and other (students, civil servants, teachers, etc.). The past history of brucellosis was based on the patient’s response to “Have you ever been diagnosed with brucellosis?” on admission. The medication history was based on the patient’s response to “Have you ever used medicines for treating brucellosis?” on admission, and divided into the usage of antibiotics and antipyretic [35]. Contact history was divided into cattle contact history and sheep contact history, because most of the residents in this area live by raising cattle and sheep [36]. Exposure method was divided into three categories: feeding animals, contact with animals’ products (slaughter, delivery, acquisition or processing, vaccination) and diet (consumption of raw unpasteurized milk or raw meat). Clinical phase was divided into acute phase (with symptoms less than 3 months), subacute phase (3–6 months), and chronic phase (over 6 months) according to the duration of symptoms [33], and the details were shown in Tables 1 and 2.

**Table 1:** Demographics, personal characteristics and clinical manifestations in 445 brucellosis patients with arthralgia.

Variables	n	(%)
Gender: men	321	72.1
Occupation		
Farmer and herdsman	263	59.1
Veterinarian	11	2.4
Processing staff	100	22.5
Other	71	16.0
Past history of brucellosis	75	16.9
Medication history: antibiotics	350	78.7
Medication history: antipyretic	336	75.5
Cattle contact history	159	35.7
Sheep contact history	305	68.5
Exposure method		
Feeding animals	277	62.2
Contact with animals’ products	13	2.9
Diet	16	3.6
Clinical phase		
Acute phase	343	77.1
Subacute phase	56	12.6
Chronic phase	46	10.3
Fever	323	72.6
Sweating	192	43.1
Fatigue	312	70.1
Spinal pain	306	68.8
Knee pain	94	21.1
Hip joint pain	76	17.1
Shoulder pain	47	10.6
Wrist joint pain	10	2.2
Sacroiliac joint pain	9	2.0
Ankle joint pain	17	3.8
Toe joint pain	31	7.0

Variables were described as No. (%)

**Table 2:** Laboratory test results in 445 brucellosis patients with arthralgia.

Variables	n	(%)
SAT: positive	435	97.8
Blood culture: *Brucella* spp.	155	34.8
ALT>40 U/L	112	25.2
AST>40 U/L	95	21.3
ALP>126 U/L for men, >136 U/L for women	101	22.7
γ-GT>58 U/L	179	40.2
CRP>5.00 mg/L	304	68.3
PCT>0.05 ng/mL	188	42.2
Neutrophil>6.3*10^9^/L	28	6.3
Monocyte>0.6*10^9^/L	98	22.0

SAT, serum agglutination test; ALT, alanine aminotransferase; AST, aspartate aminotransferase; ALP, alkaline phosphatase; γ-GT, glutamyl transpeptidase; CRP, C-reactive protein; PCT, procalcitonin.

Variables were described as No. (%)

### Statistical analysis

Continuous variables (Age, RF) were presented as median and inter-quartile range, and compared by Mann-Whitney test. Other categorical variables were presented as frequency and percentage, and the statistical significance was assessed by Chi-square test. The null hypothesis meant that there was no difference in the distribution of general characteristics between the RF positive group and the RF negative group. Multivariate logistic regression model was used to further analyze the relevant factors of the RF positivity, using input and stepwise forward methods. All reported probabilities (*P* values) were two-sided with *P* ≤0.050 considered statistically significant. Statistical analysis was performed using IBM SPSS software version 24.0.

## Results

445 brucellosis patients with arthralgia were involved in this study. There were 321 (72.1%) men and 124 (27.9%) women with the average age of 50.0[42.0, 58.0] years old. 263 (59.1%) patients were farmer and herdsman. 75 (16.9%) patients had past history of brucellosis. 305 (68.5%) and 159 (35.7%) patients were in contact with sheep and cattle, respectively. 277 (62.2%) patients had an epidemiological history of feeding animals. 343 (77.1%) patients were in the acute phase. 323 (72.6%) patients had fever and 312 (70.1%) patients had fatigue. The prevalence of spinal pain (68.8%) was the highest among various joint pains. The *Brucella* spp. was isolated from blood culture in 155(34.8%) patients. The details were shown in Tables 1 and 2.

### Distribution of RF

The average level of the RF in all 445 patients was 6.7[4.5, 11.6] IU/ml. As was shown in Table 3, 302 (67.9%) patients were in the RF negative group, with an average level of 5.4[3.7, 6.8] IU/ml and another 143 (32.1%) patients were in the RF positive group, with an average level of 16.5[12.2, 34.7] IU/ml, of which 45 (10.1%) patients were high-positive with RF >30 IU/ml and 98 (22.0%) patients were low-positive (10 IU/ml < RF ≤30 IU/ml).

**Table 3:** Distribution of RF in 445 brucellosis patients with arthralgia.

RF		n	(%)	M[P_25_, P_75_]
Negative		302	67.9	5.4 [3.7, 6.8]
Positive	10 IU/ml < RF ≤30 IU/ml	98	22.0	13.5 [11.2,16.7]
	RF >30 IU/ml	45	10.1	50.8 [38.1,83.8]

RF, rheumatoid factor.

Variables were described as No. (%), median and interquartile range.

### Characteristics of 445 brucellosis patients with arthralgia by RF level

The proportion of men in the RF positive group was higher than that in the RF negative group (*P* <0.050). And the RF positive group patients were older than RF negative patients (*P* <0.050). In addition, there were significant differences between RF negative patients and RF positive patients in the distributions of wrist joint pain and elevated CRP (*P* <0.050). However, there were no significant differences between RF negative patients and RF positive patients in the distributions of occupation, past history of brucellosis, medication history, contact history, other clinical manifestations and other laboratory indicators. The details were shown in Table 4.

**Table 4:** Comparison of general characteristics in 445 brucellosis patients with arthralgia by RF level.

Variables		RF^+^ (n=143)	RF^-^ (n=302)	Z/χ^2^	*P*
Age^a^ (year)		51.8[45.0,59.0]	49.0[41.0,57.0]	3.17	**0.002**
Gender: men		112(78.3)	209(69.2)	4.01	**0.045**
Occupation				4.32	0.228
	Farmer and herdsman	92(64.3)	171(56.6)		
	Veterinarian	1(0.7)	10(3.3)		
	Processing staff	29(20.3)	71(23.5)		
	Other	21(14.7)	50(16.6)		
Past history of brucellosis		17(11.9)	58(19.2)	3.71	0.054
Medication history: antibiotics		112(78.3)	238(78.8)	0.01	0.907
Medication history: antipyretic		114(79.7)	222(73.5)	2.02	0.155
Cattle contact history		43(30.1)	116(38.4)	2.94	0.086
Sheep contact history		104(72.7)	201(66.6)	1.71	0.190
Exposure method: feeding animals		98(68.5)	179(59.3)	3.54	0.060
Clinical phase				3.12	0.210
	Acute phase	112(78.3)	231(76.5)		
	Subacute phase	13(9.1)	43(14.2)		
	Chronic phase	18(12.6)	28(9.3)		
Fever		112(78.3)	211(69.9)	3.49	0.062
Sweating		63(44.1)	129(42.7)	0.07	0.790
Fatigue		104(72.7)	208(68.9)	0.69	0.407
Spinal pain		100(69.9)	206(68.2)	0.13	0.715
Knee pain		37(25.9)	57(18.9)	2.85	0.091
Hip joint pain		25(17.5)	51(16.9)	0.02	0.876
Shoulder pain		12(8.4)	35(11.6)	1.05	0.305
Wrist joint pain		8(5.6)	2(0.7)	10.74	**0.002**
Sacroiliac joint pain		2(1.4)	7(2.3)	0.41	0.725
Ankle joint pain		3(2.1)	14(4.6)	1.70	0.192
Toe joint pain		11(7.7)	20(6.6)	0.17	0.679
SAT: positive		139(97.2)	296(98.0)	0.29	0.733
Blood culture: *Brucella* spp.		54(37.8)	101(3.4)	0.80	0.372
ALT>40 U/L		39(27.3)	73(24.2)	0.50	0.482
AST>40 U/L		37(25.9)	58(19.2)	2.57	0.109
ALP>126 U/L for men, >136 U/L for women		38(26.6)	63(20.9)	1.81	0.179
γ-GT>58 U/L		66(46.2)	113(37.4)	3.08	0.079
CRP>5.00 mg/L		111(77.6)	193(63.6)	8.81	**0.003**
PCT>0.05 ng/mL		69(48.9)	119(40.9)	2.50	0.114
Neutrophil>6.3*10^9^/L		11(7.7)	17(5.6)	0.70	0.403
Monocyte>0.6*10^9^/L		32(22.4)	66(21.9)	0.02	0.901

RF^+^, rheumatoid factor positive; RF^-^, rheumatoid factor negative; SAT, serum agglutination test; ALT, alanine aminotransferase; AST, aspartate aminotransferase; ALP, alkaline phosphatase; γ-GT, glutamyl transpeptidase; CRP, C-reactive protein; PCT, procalcitonin.

Variables were described as No. (%) and compared using Chi-square test unless otherwise stated.

a. Age was described by median and interquartile range and analyzed by Mann-Whitney test.

### The results of multivariate logistic regression model

We included age, gender, wrist joint pain, and elevated CRP as independent variables, and positive RF as dependent variable. The multivariate logistic regression model showed (Table 5) that age, wrist joint pain and elevated CRP were positively associated with RF positivity, the OR was 1.02 (95% C.I. 1.00 to 1.04), 8.94 (95% C.I. 1.79 to 44.62) and 1.79 (95% C.I. 1.10 to 2.90), respectively.

**Table 5:** The relevant factors of positive RF in 445 brucellosis patients with arthralgia.

Factor	OR	95%CI		*P*
		Lower	Upper	
Age (year)	1.02	1.00	1.04	0.024
Gender: men	1.58	0.98	2.54	0.063
Wrist joint pain	8.94	1.79	44.62	0.008
CRP>5.00 mg/L	1.79	1.10	2.90	0.019

RF, rheumatoid factor; OR, odds ratio; CI, confidence intervals; CRP, C-reactive protein.

## Discussion

This retrospective cross-sectional analysis reported the distribution of RF, manifestations and analyzed the relevant factors of positive RF in 445 brucellosis inpatients with arthralgia. We found that nearly one-third of patients were RF positive, including 45 (10.1%) high-positive RF patients. We also found that arthralgia was mostly manifested in the spine, followed by some large joints such as knee joints and hip joints, and few patients showed pain in small joints such as wrist joints. Furthermore, the risk of positive RF was positively associated with age, wrist joint pain, and elevated CRP.

The distribution of positive RF in this study was similar to that of Zahra Ahmadinejad and colleagues, who found that 30.6% (15/49) brucellosis patients were RF positive [30]. And another case-control study [37] aimed at distinguishing brucellosis from RA found that only 8.8% of brucellosis patients were RF positive, and the average RF titer was 20.3 ± 60.6 IU/ml (RF normal range: 0–20 IU/ml). However, a clinical characteristics report [38] of brucellosis patients in Xinjiang Uygur Autonomous Region, China showed that up to 62.5% (15/24) of brucellosis patients had elevated RF. Although studies in different regions reported different distribution of positive RF in brucellosis patients, it did remind that brucellosis patients had elevated RF, which may lead to a greater possibility of misdiagnosis of brucellosis patients.

Extensive researches [23,39,40] have established that the positive rate of RF increases with age in healthy population. The elderly may have a slight increase in RF titer, and the positive rate of RF in the elderly over 75 years old can reach 25%. Similarly, the age of brucellosis patients in this study was also positively associated with the risk of positive RF. A possible explanations for this might be that the elderly have the senescence of the immune system [41], and RF is known to be a specific antibody IgG produced by immune response to autologous cells due to immune system disorders [26]. The current analysis in brucellosis patients with arthralgia found that the proportion of men in the RF positive group was significantly higher than RF negative group, this finding was also reported by Chen Liang et al. in spinal brucellosis patients [42]. However, it was contrary to previous studies in RA patients which have suggested that 80% of patients with positive RF are women [43,44]. We speculated that it might be the difference in the study population that led to the different gender distribution. Most brucellosis patients in China were men, who were more likely to develop brucellosis due to their interaction with livestock and products [1,42].

It is now well established from a variety of studies [7,45] that the most frequent complication of osteoarticular involvement in brucellosis patients are hip joint (up to 80%) and spinal joints (up to 54%), and brucellosis with peripheral skeleton involvement (wrist joint, ankle joint, toe joint, etc.) is less prevalent compared with spinal features, which is consistent with our findings. Furthermore, the results of multivariate analysis in this study showed that wrist joint pain was positively associated with the risk of positive RF. Prior studies [28,46] also noted that RA can affect any joint, and it is usually found in the wrist, knee, metacarpophalangeal and other small joints. However, this finding may be somewhat limited by the small sample size, only 8 patients with wrist joint pain. But it may help physicians to strengthen identification of positive RF patients with wrist joint pain during diagnosis and treatment, so as to avoid misdiagnosis and cause malignant outcome.

It is currently confirmed [47] that CRP also plays an important role in host defense against invading pathogens and inflammation by activating complement and enhancing the phagocytosis of phagocytes. Previous studies [22,24,25] have suggested that positive RF frequently coexists with elevated concentration of inflammatory markers, such as CRP, procalcitonin (PCT) and interleukin-6 (IL-6) in patients with Sjogren’s syndrome, systemic lupus erythematosus, RA and viral hepatitis. Likewise, elevated CRP was positively associated with positive RF among brucellosis patients in this study. We suggest that the most likely explanation is that *Brucella* spp. invades the body and causes the immune system and CRP to work together to resist pathogens.

This analysis had some limitations. Firstly, the population we studied was brucellosis inpatients, and other biomarkers for the diagnosis of RA such as Anti-cyclic citrullinated peptide antibody (CCP) were not available. Secondly, this study only reported some relevant factors of positive RF in brucellosis patients, and the causality still need to be further explored in our future research. Thirdly, we did not find any known and meaningful factors associated with high-positive RF, which may be due to the small number of patients with high-positive RF in this study population. But it also reminded us that patients with high-positive RF should also be paid attention to in future study. Despite these limitations, our study has several strengths, we contributed to evaluate the distribution and relevant factors of positive RF among brucellosis patients with arthralgia in details for the first time, which had certain clinical significance. And the sample size in this study was 445 brucellosis inpatients with arthralgia, which was a relatively sufficient data.

## Conclusion

In summary, our analysis suggested that the prevalence of positive RF in brucellosis patients with arthralgia was common and critical, nearly one-third of patients was RF positive. Elderly men brucellosis patients with arthralgia, wrist joint pain and elevated CRP were at high risk of positive RF.
